# Salmonella Aortitis in an Elderly Male, a Rare but Deadly Cause of Abdominal Pain: A Case Report

**DOI:** 10.5811/cpcem.2021.4.51408

**Published:** 2021-05-10

**Authors:** Kevin M. Yanushefski, Sukhdip Kaur, Mary Eberhardt

**Affiliations:** St. Luke’s University Health Network, Department of Emergency Medicine, Bethlehem, Pennsylvania

**Keywords:** Case report, aortitis, enteritis, salmonella, mycotic aneurysm

## Abstract

**Introduction:**

Infectious aortitis is a rare condition with mortality rates approaching 100% without surgical intervention. Symptoms and findings may be vague. Computed tomography (CT) with intravenous (IV) contrast, once the gold standard of diagnosis, may only show subtle findings. More recently, CT angiography (CTA) and magnetic resonance angiography have become the diagnostic modalities of choice.

**Case Report:**

A 58-year-old diabetic male presented to our emergency department with nausea, vomiting, diarrhea, fevers, and abdominal pain of two weeks duration. The patient had been seen just days before at another facility with the same complaints. He received an abdominal CT with IV contrast that was reported as negative and discharged with the diagnosis of gastroenteritis. He failed to improve and presented to our facility. On presentation, the patient was diaphoretic and uncomfortable. A repeat abdominal CT with IV contrast revealed a mantle of low density around the aorta. The patient was started on IV antibiotics, and a follow-up CTA of the abdomen and pelvis showed an irregular saccular aneurysm. Vascular surgery was consulted, and the patient underwent vascular reconstruction.

**Conclusion:**

Because of the high level of mortality seen in infectious aortitis and improvement in patient outcomes with surgical intervention, a high index of suspicion needs to be maintained in patients presenting with fever and chest, abdominal, and back pain, especially in the setting of risk factors and bacteremia. The clinician should be aware that the usual modality for the evaluation of abdominal pain, CT with IV contrast, may not be adequate to make the diagnosis.

## INTRODUCTION

Infectious aortitis is a rare condition most commonly related to bacteremic seeding of pre-existing intimal injury. Mortality rates approach 100% without surgical intervention.^1^–^3^ Symptoms are frequently vague and include abdominal and back pain, fevers, vomiting, and diarrhea. An initial workup in the emergency department (ED) using computed tomography (CT) with intravenous (IV) contrast may not be adequate to make the diagnosis. Computed tomography angiography (CTA) and magnetic resonance angiography (MRA) may need to be added to the diagnostic workup to attain the diagnosis.^4^,^5^ Once infectious aortitis is suspected, early initiation of broad-spectrum antibiotics and vascular surgery cons’/’;ultation are critical.^4^ Given the high mortality rate of this condition if not properly identified and treated, it is imperative that emergency physicians be aware that CT with IV contrast alone may not diagnose this deadly disease.

## CASE REPORT

A 58-year-old male presented to the ED for lower abdominal pain. The patient described two weeks of initially diffuse abdominal pain, which then localized to bilateral lower quadrants with associated nausea and vomiting. Pain was exacerbated with food. The patient also reported subjective fevers, chills, decreased appetite, and diarrhea. There were no other sick contacts at home and no recent travel. The patient denied additional symptoms. His past medical history consisted of perforated diverticulitis in 1998 with 20 centimeters of bowel resection, type 2 diabetes, and hypertension. The patient had visited another hospital a few days prior and had a CT with IV contrast of the abdomen and pelvis, which reportedly was negative, and he was discharged home.

On presentation, the patient was afebrile with the following vitals: temperature 36.4 degrees Celsius; pulse 86 beats per minute; respiratory rate 18 breaths per minute; blood pressure 162/106 millimeters mercury; and 97% oxygen saturation on room air. On physical exam, he was diaphoretic and in mild distress with dry mucous membranes. Abdominal examination was significant only for moderate bilateral lower quadrant tenderness without peritoneal signs.

The patient’s initial lab work revealed a white blood cell count of 18.52 thousand cells per cubic millimeter (mm^3^) (reference range: 4.5 thousand to 11 thousand cells/mm^3^), and a lactic acid of 2.3 millimoles per liter (mmol/L) (zero to 2.3 mmol/L). The rest of the patient’s labs were within normal limits. A CT of abdomen and pelvis with IV contrast was performed, which showed a mantle of low density surrounding the middle aorta with surrounding stranding and adjacent aortic calcification. A follow-up CTA abdomen and pelvis showed an irregular saccular aneurysm involving the anterior infrarenal abdominal aorta below the level of the inferior mesenteric artery with periaortic low density concerning for inflammatory or infectious aneurysm. The patient was initially placed on vancomycin and piperacillin/tazobactam for broad-spectrum antibiotic coverage. Vascular surgery was subsequently consulted.

On the following day the patient had excision of the infected aortic aneurysm, and reconstruction with homograft performed. Operative findings included a purulent hematoma anterior to the infrarenal aorta with surrounding inflammatory changes. The patient was found to have salmonella bacteremia. Infectious disease was consulted and changed antibiotic coverage to cefepime and then ceftriaxone based on sensitivities. After a 14-day admission, the patient was discharged home with no complications. He had a negative rheumatologic workup, a screening measure typically performed in the setting of infectious aortitis, and he was ultimately continued on six weeks of ceftriaxone through a peripherally inserted central catheter.

## DISCUSSION

Aortitis is a relatively rare condition characterized by inflammation of the tunica media or intima layers of the aorta. If inflammation is isolated to the adventitia layer then it is referred to as periaortitis.^6^,^7^ The vast majority of cases are non-infectious and are secondary to a rheumatologic etiology, the most common causes being giant cell arteritis and Takayasu arteritis.^4^–^6^ Additional non-infectious causes include rheumatoid arthritis, human leukocyte antigen B27-associated spondyloarthropathies, granulomatosis with polyangiitis, eosinophilic granulomatosis with polyangiitis, Behçet’s disease, Cogan syndrome, and sarcoidosis.^4^,^6^ Infectious causes are particularly rare, but early identification is critical given a mortality rate approaching 100% without surgical intervention.^1^–^3^

**CPC-EM Capsule**

What do we already know about this clinical entity?

*Infectious aortitis is a rare condition which can occur in the setting of bacterial gastroenteritis and is associated with high mortality rates if not recognized and treated.*

What makes this presentation of disease reportable?

*This presentation demonstrates classic symptoms of the disease in a member of the traditionally at-risk population and despite this was not initially recognized.*

What is the major learning point?

*Computed tomography alone may not be enough to identify infectious aortitis, and computed tomography angiography should be considered in the appropriate clinical setting.*

How might this improve emergency medicine practice?

*This is an important addition to the emergency physician’s differential diagnosis for febrile patients with chest and abdominal pain with potentially lifesaving consequences.*

Pathogens involved in infectious aortitis are *Staphylococcus aureus* (the most common,) non-typhoidal salmonella as seen in our patient, *Streptococcus pneumonia*, and group A streptococcus. Less common infectious causes include tuberculosis and syphilis. Prior to widespread vaccination, *Haemophilus influenza* was another common cause.^4^,^5^

Infectious aortitis is most commonly related to bacteremic seeding of a pre-existing intimal injury or atherosclerotic plaque. Additional mechanisms include spread of septic emboli to the vasa vasorum, which is often related to endocarditis, contiguous spread of local infection ultimately extending to the wall of the aorta, or direct bacterial inoculation from trauma or manipulation.^2^,^3^ Infectious aortitis is commonly associated with mycotic aneurysms although it may occur as isolated aortitis as well.^4^,^6^ Aneurysmal involvement may be secondary to seeding of pre-existing aortic aneurysm or directly caused by destruction from inflammation.^5^,^8^ Neutrophilic infiltration of the vessel wall ultimately leads to breakdown of collagen and elastin, which can then progress to a saccular aneurysm. These aneurysms rapidly progress and are more likely to rupture than non-infected aneurysms.^5^ Risk factors include male gender, age > 50, diabetes, recent history of invasive catheterization, vascular risk factors such as hypertension and atherosclerosis, particularly a history of known aortic atherosclerosis, coronary artery disease, immunodeficiency, and solid organ cancer.^1^,^3^,^5^

Classic symptoms observed in cases of aortitis are fever as well as back and abdominal pain.^9^ If there is an associated aneurysm, patients may have a pulsatile abdominal mass.^5^ Fever is present in approximately 75% of patients.^2^ Symptoms typically present indolently with average duration of symptoms of around one month described in one case series.^9^ If associated with nontyphoidal salmonella (NTS), the patient may additionally have symptoms of nausea, vomiting, and diarrhea.

Standard lab evaluation includes complete blood count, erythrocyte sedimentation rate, C-reactive protein, and blood cultures.^4^,^9^ As most cases of aortitis are non-infectious, a rheumatologic panel to screen for non-infectious etiology is also reasonable.^4^ Leukocytosis is seen in 65–83% of patients.^2^ Blood cultures are positive in the majority of cases.^7^,^9^ Of note, 5% of patients with NTS gastroenteritis ultimately develop bacteremia, of whom 40% go on to develop extraintestinal infection.^1^ About 25% of patients over 50 found to have NTS bacteremia develop additional endovascular complications.^3^

Diagnosis is primarily made radiographically with multiple modalities showing utility. Computed tomography with IV contrast was originally the gold standard and is still recommended by some sources, although CTA and MRA have largely replaced it as the imaging modalities of choice.^2^–^5^ In particular, CTA allows for early visualization of vessel wall changes that can facilitate more timely diagnosis.^10^ Magnetic resonance imaging with gadolinium has shown utility and is used in conjunction with edema weighted technique.^2^ Radiologic findings may include mural thickening, periaortic soft tissue density, vessel wall enhancement, periaortic gas, stranding, and aneurysm formation.^2^ Point-of-care ultrasound is useful in screening for abdominal aortic aneurysm, although it is limited in its utility as an imaging modality for isolated aortitis.^4^

Once infectious aortitis is suspected, early initiation of broad-spectrum antibiotics and vascular surgery consultation are crucial.^4^ Antibiotics should be continued for two to four weeks prior to surgery, as long as the patient remains hemodynamically stable, to decrease inflammation and optimize local surgical conditions prior to intervention.^1^,^2^ Even with early surgical intervention combined with antibiotics, the mortality rate of salmonella aortitis is around 40%.^9^ Surgical intervention involves extra anatomic bypass grafting or in situ graft placement.^5^ Endovascular aneurysm repair has utility as a temporizing measure in hemodynamically unstable patients with aortic rupture.^3^ Following surgical intervention, patients require a prolonged course of antibiotics, typically in the range of 6–12 weeks, although some sources recommend lifelong suppressive therapy in select cases.^1^,^2^,^3^,^5^ Clinical response and duration of treatment can be assessed through clearance of blood cultures and trending of inflammatory markers.^3^,^4^

## CONCLUSION

Infectious aortitis is a rare condition; however, given its high level of mortality and the dramatic difference in survival rates between patients intervened on surgically vs those treated medically, it should be considered in the differential diagnosis in patients presenting with fever, chest pain, and back pain, particularly in men over 50 with vascular risk factors. This is particularly true of patients found to be bacteremic. In patients specifically with NTS bacteremia following a primary gastrointestinal infection, it is important to maintain a high index of suspicion, particularly in the aforementioned high-risk group and especially when the patient fails to respond clinically to optimized medical therapy alone.
